# Human articular cartilage repair: Sources and detection of cytotoxicity and genotoxicity in photo‐crosslinkable hydrogel bioscaffolds

**DOI:** 10.1002/sctm.19-0192

**Published:** 2019-11-26

**Authors:** Cheryl Lee, Cathal D. O'Connell, Carmine Onofrillo, Peter F. M. Choong, Claudia Di Bella, Serena Duchi

**Affiliations:** ^1^ Department of Surgery University of Melbourne, St Vincent's Hospital Fitzroy Victoria Australia; ^2^ BioFab3D, Aikenhead Centre for Medical Discovery St Vincent's Hospital Fitzroy Victoria Australia; ^3^ Department of Orthopaedics St Vincent's Hospital Fitzroy Victoria Australia

**Keywords:** adult stem cells, arthritis, cytotoxic agents, tissue engineering, tissue regeneration

## Abstract

Three‐dimensional biofabrication using photo‐crosslinkable hydrogel bioscaffolds has the potential to revolutionize the need for transplants and implants in joints, with articular cartilage being an early target tissue. However, to successfully translate these approaches to clinical practice, several barriers must be overcome. In particular, the photo‐crosslinking process may impact on cell viability and DNA integrity, and consequently on chondrogenic differentiation. In this review, we primarily explore the specific sources of cellular cytotoxicity and genotoxicity inherent to the photo‐crosslinking reaction, the methods to analyze cell death, cell metabolism, and DNA damage within the bioscaffolds, and the possible strategies to overcome these detrimental effects.

## Graphical abstract

Three‐dimensional biofabrication using photo‐crosslinkable hydrogel bioscaffolds has the potential to revolutionize articular cartilage treatment. The photo‐crosslinking process may impact on cell viability and DNA integrity, and consequently on chondrogenic differentiation. In this review, we explore cellular cytotoxicity and genotoxicity inherent to the photo‐cross‐linking reaction, the methods to analyze them, and the possible strategies to overcome these detrimental effects.

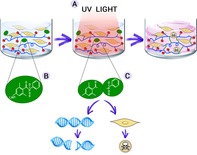

**Significance statement**

Several hurdles need to be addressed before the clinical translation of articular cartilage regeneration procedures using photo‐crosslinkable hydrogels. Cellular cytotoxicity and genotoxicity need to be identified and carefully detected to provide an indication of the safety of the repair treatment approach in patients.

## 1 INTRODUCTION

Biofabrication is the automated generation of biologically functional products with structural organization from living cells, bioactive molecules, biomaterials, and cell aggregates such as microtissues or hybrid cell‐material constructs.^1^ It has become an important tool within the regenerative medicine and the tissue engineering research field, providing new capabilities to create, for instance, implantable 3D constructs composed of biomaterials and living cells, intended as bioscaffolds. The biofabrication approach, of which the long‐term goal is to switch from nonbiological prosthesis to biological implants has generated promising results in the repair of articular cartilage.^2^ Traumatic cartilage (chondral) injuries can result in osteoarthritis, a major source of disability in the developed world.^3^ Current treatments to repair chondral lesions, which include autologous chondrocyte implantation, mosaicplasty, and microfracture, are unable to reproduce hyaline cartilage capable of sustaining shear and compressive forces associated with normal joint function. The generation of a bioscaffold, using a combination of biomaterials and cells, is a possible solution for cartilage repair.^4^ Currently, this technology has generated promising results both in vitro^5^ and in in vivo^6^ as well as in preclinical studies.^7^ Nevertheless, there are several open questions with respect to its clinical use,^8^ especially regarding the safety of the cells implanted, given the multiple sources of cytotoxicity and genotoxicity intrinsic to the bioscaffold generation process. In particular, the chemistries required to generate covalently crosslinked 3D hydrogel environments can have cytotoxic impacts on embedded cells. To our knowledge, the wider literature describing these issues has not previously been critically examined. Thus, the aim of this review is to summarize the sources of cellular damage and provide an indication on the reliable tests to be used to verify the safety of the cells implanted in bioscaffolds. Limitations of current surgical treatments for cartilage repair such as microfracture have prompted the field of cartilage regenerative medicine to integrate engineering and biological principles to promote the growth of new cartilage to replace the damaged tissue. To date, a wide range of scaffolds and cell sources have emerged toward cartilage tissue engineering, with a focus on recapitulating microenvironments present during the human body development or in the adult tissue. These microenvironments should induce the formation of cartilaginous constructs with biochemical and mechanical properties similar to the native tissue. Hydrogels have emerged as a promising scaffold material due to the wide range of properties that are possible to achieve, and the ability to trap cells within the material.^9^

## 2 PHOTO‐CROSSLINKABLE HYDROGEL BIOSCAFFOLDS FOR ARTICULAR CARTILAGE REPAIR: GENERAL OVERVIEW

Cartilage biofabrication strategies are designed to overcome the limitations of injection‐based stem cell therapies; namely, the massive cell death upon delivery caused by shear stress from the needle, poor engraftment of delivered cells and, as a consequence, limited ability to differentiate into a chondrogenic phenotype.^10^, ^11^ Rather than injecting cells directly, one alternative is to deliver stem cell laden hydrogel bioscaffolds to fill the defect. The encapsulating hydrogel has a protective effect on the cells from shear stress and mechanical cytotoxicity coming from the extrusion or the bioprinting process. The 3D microenvironment provided by the hydrogel scaffold supports cell survival and can stimulate differentiation into mature chondrocytes capable of producing their own extracellular matrix. The scaffold is typically designed to degrade over time while it is replaced by newly formed cartilage tissue arising from the cells implanted, producing a tissue which resembles the native articular cartilage. Traditional techniques to generate bioscaffolds include casting of cell‐laden hydrogel‐based materials in molds of the desired size and structure to be implanted in the defects to be treated, but also inkjet 3D bioprinting, micro‐extrusion, in situ bioprinting (Table 2). More advanced biofabrication techniques include the production of neocartilage tissue to better recapitulate the native zonal architecture of the articular cartilage, or the generation of multiphasic constructs with hybrid approaches using different materials, 3D printing techniques,^12^ or different cell sources and lineages.^13^

To achieve encapsulation, the cells are typically mixed with a liquid hydrogel solution, which is then crosslinked to form a contiguous stable network under physiological conditions.

Since the behavior of chondrocytes is, in part, mediated by the mechanical environment, matching the mechanical properties of the scaffold to that native cartilage also needs to be considered.^14^ However, the crosslinking reaction can impact on cell viability and metabolism, and consequently on chondrogenic differentiation. One of the most widely adopted crosslinking strategy uses polymers (naturally derived or synthetic) which have been modified with reactive groups (methacrylate and/or methacrylamide) which can undergo chain polymerization reactions.^15^, ^16^, ^17^ The process of protein crosslinking comprises among all chemical, enzymatic, chemo‐enzymatic, self‐assembly, ionic, thermal formation of new covalent bonds between polypeptides.^18^ These reactions allow the site‐directed coupling of proteins with distinct properties and the de novo assembly of polymeric protein networks. The chemical photo‐crosslinking process is the most investigated and the most common way to achieve a precise spatial and temporal hardening of the hydrogels. At the same time, it is the one that deserves much attention given the presence of different drawbacks that can impair cell viability. One of the main advantages of photo‐crosslinking is the rapid formation of hydrogel networks at ambient temperature under mild conditions, and the tunability of the mechanical properties. The crosslinked site is also ready to be accurately selected, because the photoinitiated polymerization takes place under light exposure and only the irradiated areas are involved in hydrogel crosslinking. The ionic/electrostatic interactions can instead achieve extremely limited mechanical strength. Moreover, the photo‐crosslinking process is the preferable choice to perform in situ bioprinting with robotic arms or handheld approaches,^19^ which is emerging as a favored bioprinting strategy during certain clinical situations when compared with conventional in vitro bioprinting. Finally, the photo‐crosslinking strategy allows to a precise temporal and spatial control compatible with the time frame in theater for surgical operations.

**Figure 1:**
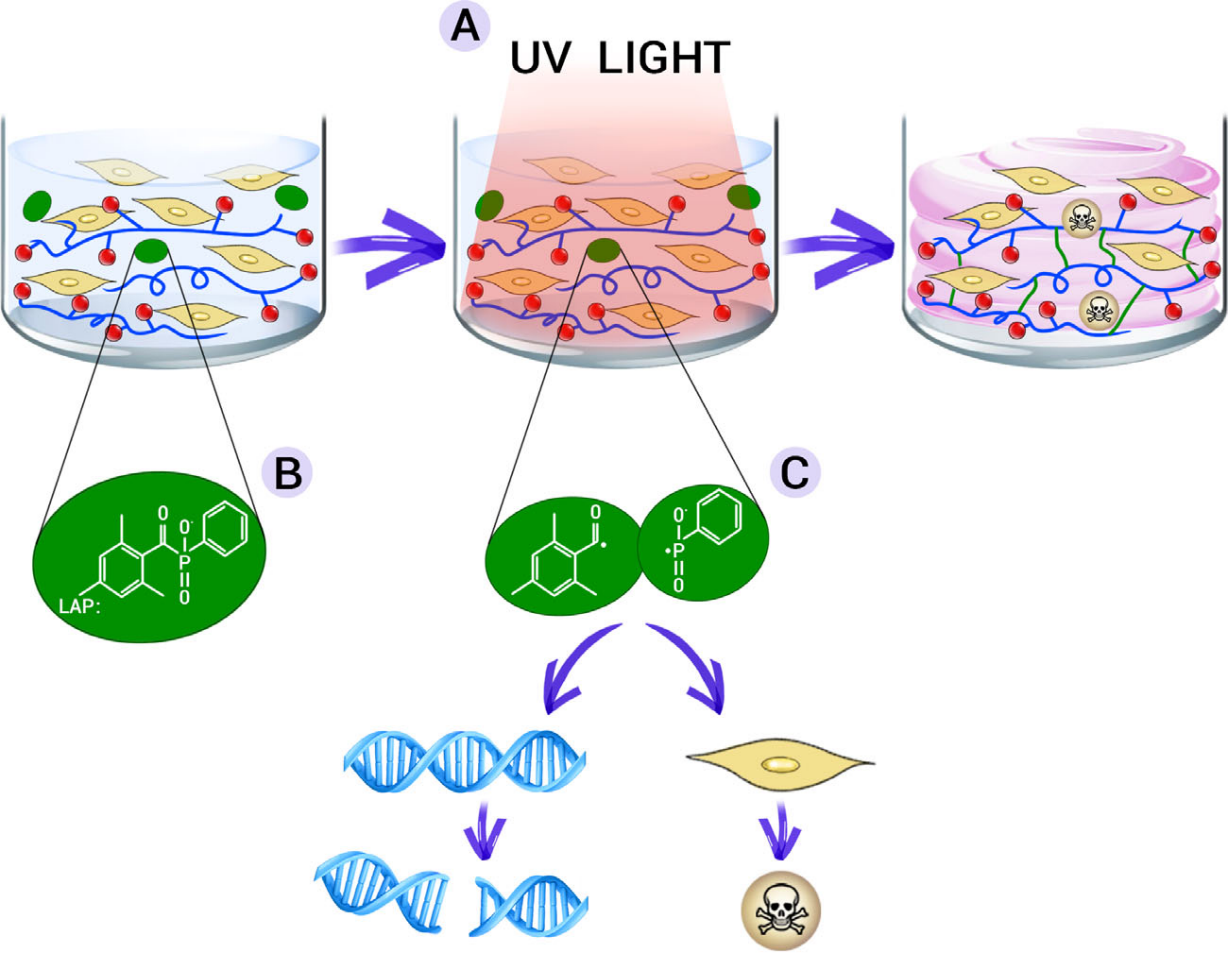
Schematic representation of the photo‐crosslinking process of a hydrogel laden with cells. Although the bio‐ink is extruded in gel form, it hardens following exposure to UV light (A). The photoinitiator molecule (eg, lithium phenyl‐2,4,6‐trimethylbenzoylphosphinate, LAP) mixed within the hydrogel (B) is cleaved and forms two free radicals (C), which are responsible for the formation of highly resistant covalent bonds between polymer chains in the hydrogel, but at the same time can lead to DNA damage and cell death

In the light‐induced crosslinking process, a photoinitiator (PI) molecule is mixed within the reactive hydrogel, and the reaction is initiated through exposure to UV or visible light of a wavelength (Figure 1). However, such photo‐crosslinking reactions can create a transiently cytotoxic environment, which may compromise cell viability and/or phenotype.^20^ Other sources of cytotoxicity inherent in the bioscaffold generation process include the shear stresses during the extrusion and the poor diffusion of nutrients or oxygen through the crosslinked hydrogels.^21^

Extended “fabrication times” involving exposure to nonphysiological conditions, such as room temperature or the lack of control of oxygen and CO_2_ levels within an enclosed bioprinting cartridge, can also have a strong impact on viability in later culture.^22^, ^23^

The clinical application of articular cartilage repair strategies will require the identification, quantification, and mitigation of such toxic effects. The scope of this review is to cover the specific sources and detection methods of cellular cytotoxicity and genotoxicity inherent to the photo‐crosslinking reaction.

Thus, in the following sections, we will examine: (a) the sources and effects of the photo‐crosslinking process on cells viability and DNA integrity; (b) the methods to analyze cell death, metabolism and DNA damage within the bioscaffold; and (c) potential solutions to overcome these detrimental effects.

## 3 PHOTO‐CROSSLINKABLE HYDROGEL BIOSCAFFOLDS FOR ARTICULAR CARTILAGE REPAIR: COMPONENTS AND PROCEDURES

The assessment of the cytotoxic and genotoxic effects of the photo‐crosslinking process requires preliminary consideration of the type of hydrogels and cells that constitute the bio‐ink and therefore the final bioscaffold. Although the type of cells may differ in their susceptibility to cyto‐ and genotoxic effects, the hydrogel itself can influence cell survival and behavior through the presence of functional groups and bioactive moieties that favor its hardening. Finally, the method of delivering the bioscaffold may influence the crosslinking conditions.

### 3.1 Sources of cells

Adult mesenchymal stem cells possess self‐renewing abilities and inherent chondrogenic properties which lend to be the elective cell type in cartilage regeneration.^24^ In particular, human adipose‐derived stem cells (hADSCs) have been incorporated into many different scaffold‐based systems and have shown promising results in cartilage tissue engineering.^25^ The two major sources of hADSCs are abdominal fat and infrapatellar fat pad (IFP).^26^ The IFP can be opportunistically harvested during routine surgical procedures such as knee arthroplasty or arthroscopy, and is known to have high chondrogenic potential.^27^ Bone marrow‐derived stem cells share common properties with hADSCs but are limited due to low tissue availability and cell number, and inferior chondrogenic potential compared with IFP‐derived stem cells.^28^ Mature chondrocytes instead possess poor replicative capacity and are prone to dedifferentiation, thus are not ideal for the purpose of cartilage regeneration.^29^ hADSCs are currently under consideration for several clinical applications (https://www.clinicaltrial.gov/),^24^, ^30^ and they are known to accumulate DNA damage and undergo senescence during in vitro cultivation, so stem cell preparations already undergo rigorous testing in order to ensure safety for the recipient.^31^ These cells are also known for their resistance to chemotherapeutics drugs and their promising use as drug delivery tools, especially for bone‐derived tumors.^32^, ^33^, ^34^ Another source of chondrogenic cells for articular cartilage repair are nasal chondrocytes. In fact, hyaline‐like cartilage tissues, engineered from autologous nasal chondrocytes, have shown promising results in a human clinical trial in patients with traumatic full thickness cartilage lesions.^35^

### 3.2 Photo‐crosslinkable hydrogels and crosslinking process

**Figure 2:**
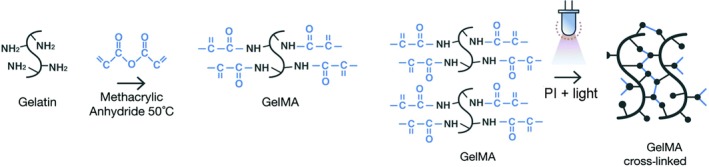
Schematic representation of the methacrylation of gelatin to form GelMa. Functional side chains of the GelMa molecule can be photo‐crosslinked by adding a specific photoinitiator (PI) and light irradiation, to form a network contributing to the stiffness of the resulting scaffold. *Source*: Adapted from Caballero Aguilar et al,^39^ reproduced with permission of Royal Society of Chemistry via Copyright Clearance Center

The ideal hydrogel for cartilage regeneration is one that resembles the natural extracellular matrix of cells to support cell survival and differentiation and thus to form functional articular tissue.^36^ Natural hydrogels such as gelatin display high biocompatibility and biodegradability.^37^ Gelatin is composed of hydrolyzed collagen and retains abundant Arginine‐Glycine‐Aspartate sequence motifs which serve as cell attachment sites, and it contains matrix metalloproteinase sensitive degradation sites. These bioactive motifs facilitate cell adhesion, proliferation, and differentiation via integrin‐mediated cell adhesion and cell‐mediated enzymatic degradation. However, in order to be used as a biomaterial, gelatin needs to be modified to gain irreversible crosslinking, necessary strength, and precise mechanical tunability.^38^ This problem can be overcome by the addition of functional groups such as methacrylate/methacrylamide, which can be crosslinked after the activation of a PI, to form gelatin methacryloyl (GelMa) (Figure 2). This achieves mechanical tunability while retaining the biocompatibility of gelatin, making GelMa the most popular choice of hydrogel in hydrogel‐based cartilage repair.

Similarly, hyaluronic acid, which supports cell differentiation along the chondrogenic lineage, can be modified to form cross‐linkable methacrylated hyaluronic acid (HAMa) which, when combined with GelMa to form GelMa‐HAMa, increases the mechanical properties of tissue engineered articular cartilage.^40^, ^41^ Nevertheless, GelMA itself does not contain all the required biofunctionality to support chondrogenic differentiation of the stem cells. As such, HAMa is acting also as one of the biological molecules to enhance the chondrogenic potential of a bioscaffold, together with other additives such as methacrylated chondroitin sulfate (CS‐Ma), or even combination of both HaMA and CS‐Ma in GelMA.^42^

Synthetic hydrogels such as polyethylene glycol (PEG) exhibit limited biocompatibility compared with natural hydrogels but are useful for their superior mechanical properties.^43^ Introduction of acrylate functional groups forming polymerizable PEG diacrylate (PEGDA) as well as the addition of other moieties to improve the biological properties has propelled PEGDA to become a popular hydrogel choice in cartilage tissue engineering.^44^, ^45^, ^46^

**Figure 3:**
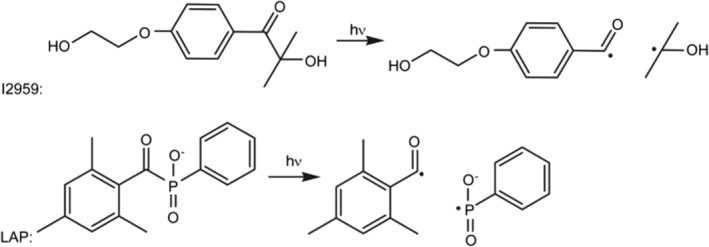
Examples of two commonly used PI molecules: Irgacure 2959 (2‐hydroxy‐4′‐(2‐hydroxyethoxy)‐2‐methylpropiophenone) and LAP (lithium phenyl‐2,4,6‐trimethylbenzoylphosphinate), generating two free radicals after cleavage with UV light (hv). These two free radicals will then attack functional groups on the hydrogel to initiate the polymerization reaction. *Source*: Adapted from Fairbanks et al^49^

Hydrogel crosslinking can be achieved via a photoinitiated but also enzymatic system. Enzymatic crosslinking commonly utilizes transglutaminases, tyrosinases, or peroxidases to catalyze the formation of highly resistant covalent bonds between polymer chains.^47^ The main drawbacks are the instability of some of the enzyme types, especially transglutaminases and tyrosinases, and the limited mechanical properties of the gels formed. As described above, photo‐crosslinking has been demonstrated to provide excellent temporal and spatial control over the process hence allows greater control of the mechanical properties of the resultant matrix compared with the indirect enzymatic process.^48^ The reaction involves the usage of light of a specific wavelength to strike the PI, which in turn is cleaved into two free radicals (Figure 3). One or both of these free radicals then radicalize a nearby reactive functional group (methacrylate or methacrylate) which propagates the polymerization chain reaction.

As the reaction proceeds the number of the crosslinks in the system increases exponentially resulting in a biopolymer network linked through polymethacryloyl chains.^43^ The degree of crosslinking and therefore the degree of mechanical stiffness is a result of PI type and concentration, light intensity, wavelength, exposure time, and degree of methacrylation.^50^ Although the photo‐crosslinking process is efficient and is somewhat controllable, it presents three major potential sources of cellular toxicity: generation of free radicals, exposure to the PI molecule itself, and exposure to light. Ultimately, the toxicity introduced by the photo‐crosslinking process must be minimized to achieve a crosslinked hydrogel with optimal mechanical stiffness, maximal cell viability, and minimal DNA damage.

## 4 SOURCES OF CYTOTOXICITY AND GENOTOXICITY

### 4.1 Light source and irradiation

The photo‐crosslinking process commonly employs an ultraviolet (UV) light source which is by itself a potential source of cytotoxicity, due to UV induced apoptosis and most importantly, genotoxicity when DNA damaged cells are not eliminated. Long‐wave UV (A, 315‐400 nm) is widely accepted as a mutagen owing to its ability to induce cellular DNA damage, and shortwave UV (B, 280‐315 nm) irradiation can lead to DNA base lesions such as cyclobutane pyrimidine dimers (CPDs), and pyrimidine 6‐4 pyrimidone photo‐products.^51^ Higher wavelength UV‐A (315‐400 nm) induced cytotoxicity occurs mostly via indirect mechanisms, whereby cellular chromophores act as photo‐sensitizers to generate reactive oxygen species (ROS) which causes insult to proteins, lipids and DNA, the main lesion being the oxidized base 8‐oxo‐7,8‐dihydroguanine (8‐oxoG).^52^, ^53^ Similarly to CPDs, 8‐oxoG can pair with adenine and cause a guanine:cytosine to thymine:adenine transversion, but can also result in DSBs if inserted during DNA replication.^54^ Other studies identified an action spectra to determine cell killing and mutations by monochromatic ultraviolet and visible radiations (254‐434 nm) in human epithelial cells.^55^

More recently, the cytotoxicity of UV‐A1 radiation was tested in human mesenchymal stem cells and data show that a prolonged 2‐hour exposure to high intensity (370 ± 5 nm; 788 kJ/m^2^) in the absence of any PI, results in a significant reduction of cell viability of up to 50% compared with cells exposed to visible light only.^56^ Nevertheless, it has also been demonstrated that low‐dose and long‐wave UV‐A light do not affect their gene expression, making ideal cells candidate for their low susceptibility to cytotoxic and genotoxic effects derived from a photo‐crosslinking reaction.^57^

**Figure 4:**
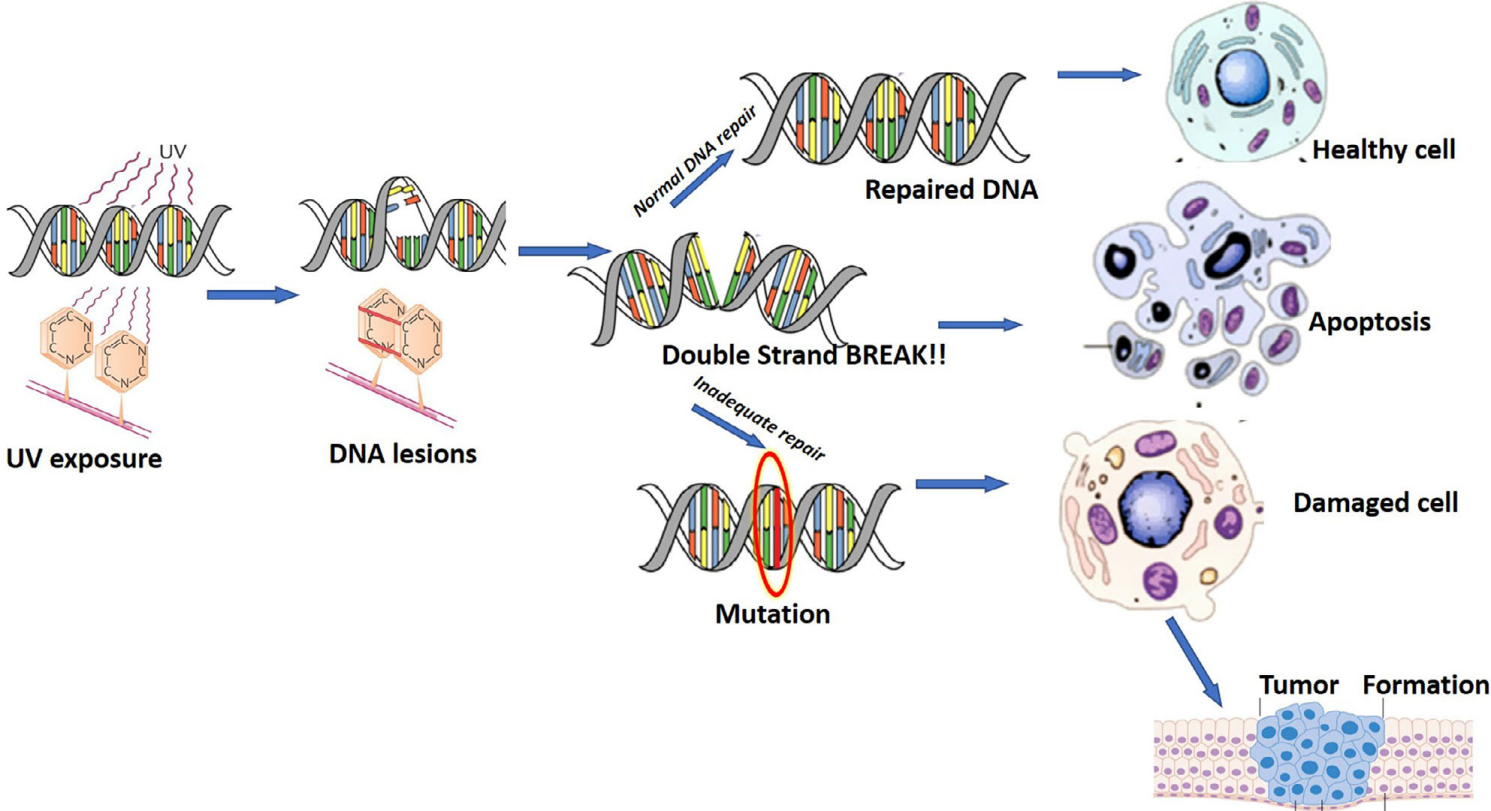
Schematic representation of UV induced DNA lesions and potential detrimental outcomes resulting from inadequate repair

Double‐stranded breaks (DSBs), which are considered the most deleterious type of DNA damage, can be caused as a direct result of these lesions and indirectly through the production ROS.^58^ Inbuilt cellular mechanisms to avoid mutagenesis include DNA repair, apoptosis or cell cycle arrest. However, should these mechanisms fail, these genomic lesions can result in tumor formation^59^, ^60^ (Figure 4).

### 4.2 Photoinitiator molecules

**Figure 5:**
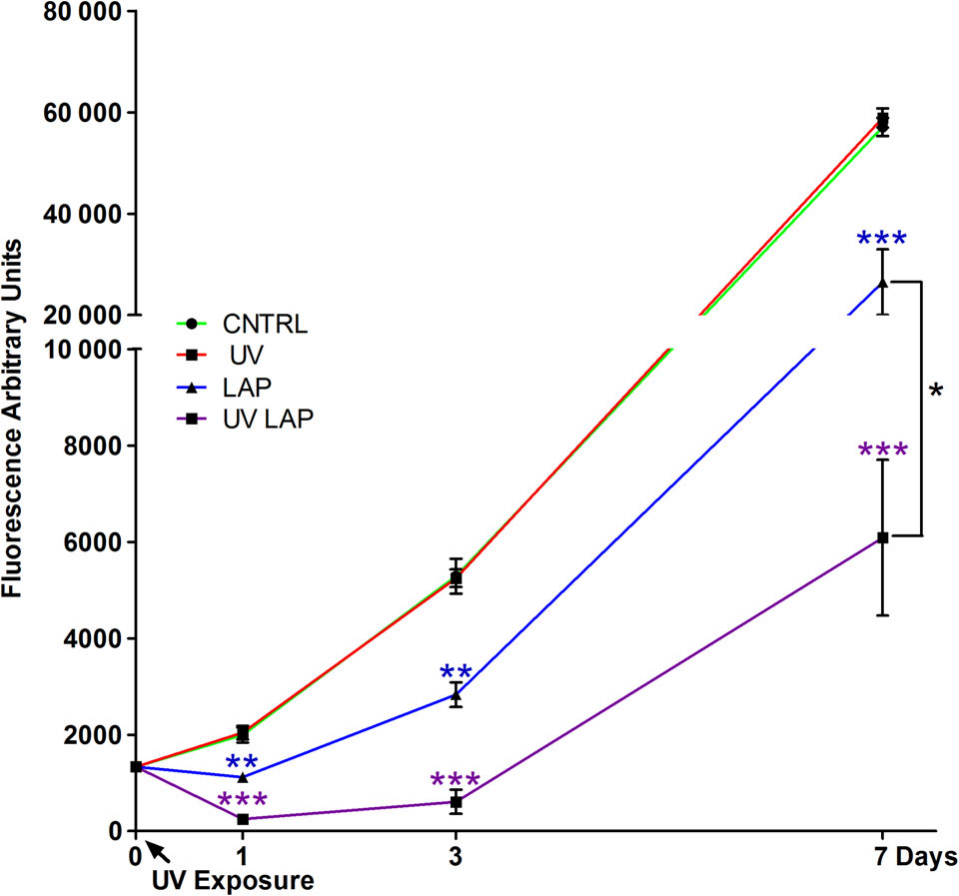
Cell cytotoxicity induced by the photoinitiator LAP and UV light irradiation at 365 nm with an irradiance of 700 mW/cm^2^ for 10 seconds. Human adipose‐derived stem cells (hADSCs) cultured in 2D and assayed along 7 days in culture with a metabolic test (Cell Titer‐Blue) to measure the cytotoxicity induced by cell exposure to UV light alone (UV), LAP on its own (LAP), and UV exposed LAP (UV LAP) compared with untreated cells (CNTRL). Error bars represent SEM between three replicates. The calculated statistical significance was obtained by unpaired *t* test and calculated vs CNTRL. At day 7 statistics is calculated also for UV LAP vs LAP. In all graphs stars represents * is p ≤ 0.05; ** is p ≤ 0.01; *** is p ≤ 0.001; not significant (n.s.) is p > 0.05. *Source*: Used with permission (http://creativecommons.org/licenses/by/4.0/) from Duchi et al^62^

The optimal PI is one that generates phase transformation of hydrogels from gel to solid to withstand required compressive forces for the target tissue while remaining minimally toxic to the encapsulated cells. The PI itself has intrinsic toxic effects although this varies between PIs and can be minimized by selecting the lowest practicable concentration; although reducing the PI concentration necessitates an increase to the light exposure time or intensity, it needs to achieve equivalent mechanical properties.^50^ The toxicity of PI molecule relates to its chemical structure, especially its hydrophobicity, which increases its potential to cross the cellular membrane. A comparative study between three PIs, Irgacure 2959 (2‐hydroxy‐4′‐(2‐hydroxyethoxy)‐2‐methylpropiophenone), Irgacure 184 (1‐hydroxy‐cyclohexyl‐phenyl‐ketone), and Irgacure 651 (2,2‐dimethoxy‐1,2‐diphenylethan‐1‐one), found that Irgacure 651, the most nonpolar molecule of the PIs, exhibited the highest cytotoxicity whereas Irgacure 2959, which has a relatively polar structure, was the least toxic.^61^ Several studies have analyzed the individual effects of the different components of the photo‐crosslinking process on cell viability in 2D by exposing cells to UV irradiation, PI alone, and PI with UV irradiation.^20^, ^61^, ^62^ Table 1 summarizes the intrinsic toxicity of different PI molecules. However, to be comparable, the susceptibility of the cell type used, the concentration of the PIs, the exposure times to light and the techniques used to generate the bioscaffolds must be carefully taken into consideration, but this was beyond the principal scope of the review. Furthermore, as PIs vary in their crosslinking efficiency, the concentration required for hydrogel hardening differs between PI types. For this reason, toxicity and efficiency should both be considered in choosing the ideal PI. As an example of intrinsic toxicity, the LAP (lithium phenyl‐2,4,6‐trimethylbenzoylphosphinate) PI molecule has been studied in relation to cellular survival: in a 2D culture of hADSCs, the survival of cells was highly affected by exposure to UV light at 365 nm, with an irradiance of 700 mW/cm^2^ for 10 seconds in combination with LAP, as well as to LAP alone, but not to UV light itself (Figure 5). This was one of the main aspects that prompted the exploration of protective elements for the cells, such as, for example, their encapsulation in hydrogel or a spatial separation from the PI (ie, coaxial printing techniques that can separate cells from direct contact with the PI). These strategies have been demonstrated to significantly reduce the cytotoxic effect of these chemicals.

**Table 1:** Common photoinitiators (PIs) used in hydrogel photo‐crosslinking and summary of toxicity measured in 2D cell monolayer without light irradiation

PI	Peak absorbance wavelength	Toxicity	References
Irgacure 2959 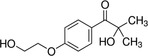	365 nm UV‐A	Between 0.05% and 0.1% viability of hBMSCs and bovine chondrocytes ≈90%	20, 61, 63
VA086 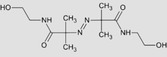	370‐405 nm UV‐A	Up to 1.4%, bovine chondrocyte viability >90%	63
Camphorquinone 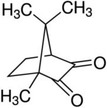	400‐520 nm VIS	hGF treated with 0.5‐2.5 mM CQ had similar viability to untreated cells	64
LAP 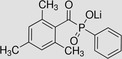	365‐490 nm VIS	At 0.1% hADSCs demonstrated a significant reduction in viability	62
Eosin Y‐TEA 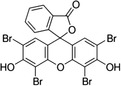	470‐550 nm VIS	0.1 mM EY and 1.5%v/v TEA are “very toxic” to hMSCs	65
Rose Bengal (RB) 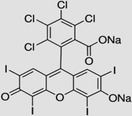	VIS	At 0.01%, rBMSCs viability is ≈80%, but at 0.1% this falls to ≈30%	66

Abbreviations: Camphorquinone, 2,3‐bornanedione; hADSC, human adipose‐derived stem cells; hBMSC, human bone marrow‐derived stem cells; hGF, human gingival fibroblasts; hMSCs, human mesenchymal stem cells; Irgacure 2959, 2‐hydroxy‐4′‐(2‐hydroxyethoxy)‐2‐methylpropiophenone; LAP, lithium phenyl‐2,4,6‐trimethylbenzoylphosphinate; rBMSC, rabbit bone marrow‐derived stem cells; Rose Bengal, 4,5,6,7‐tetrachloro‐2′,4′,5′,7′‐tetraiodofluorescein; TEA, triethylamine; VA‐086, 2,2′‐azobis[2‐methyl‐*N*‐(2‐hydroxyethyl)propionamide].

### 4.3 Free radicals

As discussed above, photoinduced free radicals are highly reactive species, chosen for their ability to trigger a radical polymerization reaction. However, they can also interact with double bonds within cellular components such as membranes, proteins, and DNA, thus threaten cell viability, metabolism, and DNA integrity.

The toxicity of free radical can arise through direct effects, as well as indirect effects, such as the formation of ROS upon reaction of a free radical with the environmental oxygen.^67^ Oxidative degradation of lipids which constitute the cell and mitochondrial membranes produce toxic aldehyde end products such as 4‐hydroxynonenal (4‐HNE). 4‐HNE is particularly cytotoxic and mediates this effect through depletion of glutathione, a potent antioxidant that has a role in mitochondrial redox reactions, and the formation of mitochondrial protein adducts.^68^ The subsequent disruption of mitochondrial function activates intrinsic apoptotic pathways, although it should be noted that at very high concentrations, acute cell death by necrosis can occur.^69^ In terms of genotoxicity, free radical‐induced DNA damage can take the form of base lesions, damage to the sugar moiety, tandem lesions, DNA‐protein crosslinks, single, and double strand breaks.^70^ Of the bases, guanine is most susceptible to oxidative stress leading most commonly to the formation of 8‐oxoG lesions as discussed above, but also to other products such as imidazolone and spirodihydantoin.^71^ These different oxidative lesions will result in different transversion mutations unless adequate DNA base excision repair mechanisms are used. If resulting mutations occur within critical regions of the genome responsible for regulating cell proliferation such as tumor suppressor genes or oncogenes, dreaded malignant transformation of cells the can arise.^72^ DSBs can be repaired by nonhomologous end joining, an error prone mechanism that often introduces mutations.^73^ Direct induction of DSBs occurs through a reaction between hydroxyl radicals and the DNA molecule producing single strand breaks. When two closely opposed single strand breaks, commonly referred to as “clustered damages,” form, the molecule cannot be resealed thus converts into a DSBs.^74^ In addition to breaks, repair of other clustered DNA lesions by simultaneous base excision repair can result in single strand breaks which can convert to DSBs.^75^ Finally, during DNA replication, the presence of single strand breaks or other DNA lesions such as interstrand crosslinks or DNA‐protein crosslinks can hinder the normal replicative process leading to a collapse of the replication fork and DSBs formation.^76^ As such, the formation of less harmful lesions such as base lesions described in the paragraph above have the potential to form these highly risky DSBs.

The reduction in cell viability due to cytotoxic effects of free radical photoinitiation has been well characterized across different PI types. Fedorovich et al demonstrated that the combination of UV light with Irgacure 2959 resulted in the highest cytotoxicity compared with the two modalities alone.^20^ Similar results were generated with LAP and RB indicating that the PI toxicity is drastically exacerbated by photoactivation.^62^, ^65^, ^66^

More concerning than cell death is the damage to cells that survive despite free radical induced toxicity. Evidently, the significant drop in cell metabolic activity immediately after high intensity UV crosslinking, and the progressive decline over the following week, suggests that engendered free radicals from light‐induced PI degradation causes irreparable damage to cellular processes.^50^ O'Connell et al demonstrated in fact that although metabolic activity declined, cell survival remained high (>90%) which raises concern that damaged cells could contain DNA‐base lesions. As discussed earlier, depending on the genes where these lesions occur, tumor formation within the generated bioscaffold could result, rendering this technology unsafe for clinical application.

## 5 TECHNIQUES FOR ANALYSIS OF CYTOTOXICITY AND GENOTOXICITY

The rate of survival and safety of cells in the bioscaffolds need to be carefully evaluated through an assay that can detect markers of live, dead, and damaged cells, and that can penetrate the crosslinked hydrogel. The conventionally used assay LIVE/DEAD viability kit (e.g., Thermo Fisher Scientific Inc., Waltham,MA, USA, https://www.thermofisher.com/au/en/home/brands/molecular‐probes/key‐molecular‐probes‐products/live‐dead‐viability‐brand‐page.html) utilizes green‐fluorescent Calcein‐AM and red‐fluorescent Ethidium Homodimer which indicate intracellular esterase activity in live cells and loss of plasma membrane integrity in dead cells respectively. Direct visualization of the cells by fluorescent imaging provides information approximately cell morphology and behavior within the hydrogel.^77^ Furthermore, spatial information regarding the position of live and dead cells within the bioscaffold can be obtained and this can inform at what extent the distance of cells from the light source affects cell survival.^78^ Meanwhile, quantitative information can be obtained by the counting of imaged cells. Alternatively to microscopic imaging, flow cytometry of single cells suspension from the bioscaffold can provide quantitative measurements.^79^ Table 2 highlights in the most recent literature the frequent use of LIVE/DEAD assay performed on bioscaffolds for cartilage repair to assess cytotoxicity.

**Table 2:** Summary of the most relevant cell viability studies following photo‐crosslinking of different hydrogels under various conditions for cartilage repair treatments

Hydrogel	Photoinitiator (w/v)	Cross‐linking conditions	Cell type	Viability assay	Results	3D bioscaffolds generation	References
PEGDA, HAMa, CSMa with RGD/RDG modifications	Irgacure 2959 (0.05% w/v)	UV light, 3.5 mW/cm^2^, 5 min	Primary bovine chondrocytes	LIVE/DEAD	All conditions supported cell viability. 86% of encapsulated cells were alive, and the presence of RGD did not enhance the chondrocyte viability	Casting of cells mixed with hydrogel in cylindrical molds	80
GelMa (mGL)	LAP (0.15% w/v)	430‐490 nm VL, 1400 mW/cm^2^, 4 min	hBMSCs	LIVE/DEAD MTS assay	92% viability after 90 days based on live/dead staining. MTS assay demonstrated significantly higher cell metabolic activity compared to non‐cross‐linked agarose constructs	Casting of cells mixed with hydrogel in silicon molds and then punched into 5 mm × 2 mm cylinder	81
PEGDA	Irgacure 2959 (0.05% w/v)	UV lamp (model B‐100AP; UVP) 4.5 mW/cm^2^ Photocrossinking was carried out simultaneously with printing (approx. 108 s) or for 10 min post‐printing	Human articular chondrocytes	LIVE/DEAD	Cells viability with simultaneous photopolymerization was 89.2% ± 3.6%, compared with 63.2% ± 9.0% in post‐printing polymerization	HP Deskjet bioprinter in bovine osteochondral plugs	82
GelMa‐HAMa	LAP (0.1%)	365 nm, 700 mW/cm^2^, 10 s	hADSCs printed in a monoaxial configuration or separated from the PI using coaxial printing	LIVE/DEAD	The monoaxial group showed a viability decrease by 30% compared with coaxial printing at day 10	Handheld bioprinting with Biopen device	62
GelMa	Irgacure 2959 (0.5%)	365 nm, 10, 100, and 700 mW/cm^2^ for 316, 100, and 37 s, respectively	hADSCs	LIVE/DEAD CellTiter‐Blue cell viability assay	At 100 mW/cm^2^ live/dead shows >90% viability. Metabolic tests show a significant decrease in the 700 mW light intensity group compared with the other groups	Casting of cells mixed with hydrogel in PDMS cylindrical molds	50
Alg‐GelMa, Alg‐GelMa‐HAMa, Alg‐GelMa‐CSMa‐HAMa	Irgacure 2959 (0.05% w/w)	UV light, 6 mW/cm^2^, 30 s	hBMSCs	LIVE/DEAD	All groups showed >85% viability 3 hours after printing but it decreases over 3 wk. The Alg‐GelMa‐CSMa‐HAMa suffered the largest decline in viability	Custom coaxial dispensing system	83
Gelatin furfurylamine	Rose Bengal (0.05% and 0.1%)	Vis light lamp, 2 min	Rabbit BMSCs	WST assay	Viability in 0.05% RB group was 87.3% at 24 hours, 55.8% on day 3% and 44.1% on day 7 after light exposure, whereas cells in the 0.1% RB group only showed 64% viability at 24 hours	Casting of cells mixed with hydrogel	66
PLLA‐PEG 1000and PDLLA‐PEG 1000	LAP (0.3%)	VL lamp 395 nm, 2 min	hBMSCs	LIVE/DEAD	Cell viability at day 7 was >85% in all groups	Cells pellet and hydrogels pipetted to fill multiple circular 5 mm diameter 2 mm height molds punched out of silicone rubber	84
Methacrylated alginate at varying levels of methacrylation (8%–25%)	Irgacure 2959 (0.05% w/v)	365 nm, 10 min	Primary bovine chondrocytes	LIVE/DEAD, MTS assay	Live/dead revealed >80% cell viability in all groups and MTS showed no significant differences	Casting of cells mixed with hydrogels in 24 well tissue culture plates	85
Methacrylated alginate	Irgacure 2959 (0.5% w/v) or VA‐086 (0.5%)	365 nm, 5 and 10 min	Primary bovine chondrocytes	LIVE/DEAD	Viability of the VA‐086 group remained over 90% at both time points and was similar to cells exposed to UV without the PI, whereas the Irgacure group fell to <20% after 5 min	Pouring of cells within hydrogel between two silanized glass plates spaced 1 mm apart	63
Styrenated gelatin	Camphorquinone (0.1%w/w)	Visible light 400‐520 nm, 2 min, 800 mW/cm^2^	Rabbit primary articular chondrocytes	MTT assay	Only 26% of viable cells were recovered from the hydrogel immediately after cross‐linking. This percentage decreases after 7 days	Cell–gelatin mixture casted in disk‐shaped gelatin hydrogel was approximately 2 mm in thickness, and that it contained 3 × 10^5^ cells	86
GelMa or PEGMA (BioINK) Hydrogel/PCL	Irgacure 2959 (0.05%)	UV light, 15 min	Porcine BMSCs	LIVE/DEAD	Analysis immediately after printing demonstrate >70% viability with no significant difference between groups	Casting in molds and bioprinting with 3D bioplotter RegenHU	87
GelMa/Col	Irgacure 2959 (0.05‐0.5 wt%) Ru/SPS (0.2/2 to 2/20 mM/mM)	UV light, Vis light 3‐100 mW/cm^2^, 15 min	hBM‐MSCs HACs	LIVE/DEAD Alamar blue	Even at low UV photoinitiator (I2959) concentrations (0.05 wt%), increasing UV irradiation dosage (30‐50 mW/cm^2^) causes a decrease in cell viability for both human articular chondrocytes and MSCs	BioScaffolder extrusion 3D bioprinting	88

Abbreviations: Alg, alginate; CSMa, chondroitin sulfate methacrylate; GelMa, gelatin methacrylate; hADSC, human adipose‐derived stem cells; HAMa, hyaluronic acid methacrylate; hBMSC, human bone marrow‐derived stem cells; Irgacure 2959, 2‐hydroxy‐4′‐(2‐hydroxyethoxy)‐2‐methylpropiophenone; LAP, lithium phenyl‐2,4,6‐trimethylbenzoylphosphinate; MTS, tetrazolium reduction assay; MTT, 3‐(4,5‐dimethylthiazol‐2‐yl)‐2,5‐diphenyltetrazolium bromide; PDLLA‐PEG 1000, poly‐d,l‐lactic acid/polyethylene glycol/poly‐d,l‐lactic acid; PEGDA, polyethylene glycol diacrylate; PLLA‐PEG 1000, poly‐l‐lactic acid/polyethylene glycol/poly‐l‐lactic acid; RDG, Arginine‐Aspartate‐Glycine; RGD, Arginine‐Glycine‐Aspartate; Rose Bengal, 4,5,6,7‐tetrachloro‐2′,4′,5′,7′‐tetraiodofluorescein; WST, 2‐(2‐methoxy‐4‐nitrophenyl)‐3‐(4‐nitrophenyl)‐5‐(2,4‐disulfophenyl)‐2H‐tetrazolium.

Other cell viability assays take advantage of cellular metabolic activity to estimate the number of viable cells in a sample. These include MTT (3‐(4,5‐dimethylthiazol‐2‐yl)‐2,5‐diphenyltetrazolium bromide), WST (2‐(2‐methoxy‐4‐nitrophenyl)‐3‐(4‐nitrophenyl)‐5‐(2,4‐disulfophenyl)‐2H‐tetrazolium), and Resazurin reduction assays, the latter commonly made commercially available as CellTiter‐Blue Cell Viability Assay (eg, Promega Madison, WI 53711 USA, https://www.promega.com.au/products/cell-health-assays/cell-viability-and-cytotoxicity-assays/celltiter_blue-cell-viability-assay/?catNum=G8080). Viable cells with active metabolism reduce the substrate to form a fluorescent product that can be detected and quantify by a plate reading spectrophotometer.^89^ Although this method is convenient owing to the ability of plate readers to analyze multiple wells at the same time, it does not enable the direct visualization of cells as by the LIVE/DEAD imaging. Despite its limited use in the literature, the combination of LIVE/DEAD kit with the metabolic assays could provide a more rigorous characterization of cellular viability (Table 2). Although there are many studies characterizing cell survival or the cytotoxic effects of UV light in bioscaffolds, there is limited evidence in the literature for the genotoxic effects such as the detection of double‐stranded DNA breaks or any of the lesions described above. The detection of the DNA integrity is necessary to assess the risk of tumorigenesis. However, although there are a multitude of techniques to analyze cells in a 2D monolayer, only one study has evaluated DNA damage in cells encapsulated within a 3D hydrogel scaffold. This study analyzed the genotoxic effects of photo‐crosslinking by utilizing P53‐binding protein 1 (p53BP1) as a marker of DNA damage.^20^ P53BP1 is a tumor suppressor which localizes to DSBs for DNA repair and can be detected and visualized through immunohistochemistry or western blot techniques.

Another marker of DNA damage, more specific for DSBs, is the phosphorylated form of the histone protein H2AX (pH2AX). pH2AX foci form in a 1:1 ratio with DSBs and are responsible for the recruitment of DNA repair proteins to the site of the lesion.^90^ Immunohistochemistry can be used to detect and quantify pH2Ax foci.^91^ Nevertheless, a protocol for the use of this technique in 3D bioscaffolds has not been established and its efficacy may be limited by poor antibody penetration through the cross‐linked hydrogel and background staining of the hydrogel. Alternate strategies which can be explored for the detection of these protein marker are western blotting of protein extracted from the 3D matrix and flow cytometry of single cells suspension from the scaffolds. Both this techniques are more specific than immunohistochemistry, overcome the complication of background staining, and allow quantification of the total amount of pH2AX in a cell population but foregoes 3D spatial information afforded by microscopy.^92^, ^93^

An alternative genotoxicity assay which could be adapted to analyze bioscaffolds is the comet assay, a microgel electrophoresis technique where cells with single and DSB migrate toward the anode in the shape of a comet.^94^ The degree of migration or length of the comet tail is proportional to the degree of DNA damage. This assay has been used across various cell types including mesenchymal stem cells. Although this technique has been used for analysis of cells in tissue engineered skin,^95^ it has not been used in photoencapsulated cells and would require a protocol to extract the cells from the scaffold without inflicting further DNA damage. As such, further investigation to develop a protocol for the detection of DSBs in cells within bioscaffolds is required.

## 6 STRATEGIES TO REDUCE CYTOTOXICITY

Both cytotoxicity and genotoxicity can be minimized by targeting the choice and the concentration of the PI or alternatively by the separation of cells from free radicals.

### 6.1 Photoinitiator choice and concentration

PI choice is typically based upon intrinsic properties such as their absorption spectra and its solubility limit in water. The efficiency of a PI at a wavelength of light is then determined by (a) its molar absorptivity at that wavelength, (b) the quantum yield of photolysis (the fraction of absorbed photons which produce free radicals), and (c) the PI efficiency (the ratio of initiation events to radicals generated). Unfortunately, a lack of such intrinsic data in the literature to date has severely hampered a rational comparison of relevant PIs.

The most commonly used PI in GelMa crosslinking is Irgacure 2959 which has been acclaimed for its water solubility and relatively low cytotoxicity.^61^ Irgacure 2959 has a peak absorption of 260 nm and although it can be activated at a higher wavelength of 365 nm, it has a cross‐linking time that is too long for clinical application as the extended period of UV exposure poses a risk of cytotoxicity.^96^

Across the literature, there are reports of PI concentrations ranging from 0.05% to 0.5% w/v with little consensus on the optimal concentration for cartilage tissue engineering.^61^, ^97^ Arguably, the optimal concentration will vary for the hydrogel being cross‐linked and the susceptibility of the cell type. Furthermore, Bartnikowski et al suggests that the number of reactive crosslinkable side chains on the monomers, termed degree of functionalization (DoF), should be accounted for in determining an optimal PI concentration.^98^ Chondrocytes encapsulated in “low DoF GelMA” displayed lower cell viability compared with “high DoF GelMA” following UV crosslinking with Irgacure 2959.

VA086, LAP, and Eosin Y are promising candidates. VA086 has a peak absorption of 370 nm but has been shown to crosslink hydrogels in an “ultrafast fashion” under illumination at 405 nm for only 10 seconds.^61^, ^99^ It has also been shown to be less cytotoxic than Irgacure 2959 to bovine articular chondrocytes following UV irradiation.^63^

More recently, LAP which has a peak absorbance of 385 nm but permits photo‐crosslinking at longer wavelengths, broaching the visible spectrum (405 nm), has gained popularity. It has higher water solubility compared with Irgacure 2959, thus permits cell encapsulation in PEGDA at lower PI concentrations and shorter light exposure times while maintaining >95% viability of human neonatal fibroblasts.^49^ In a comparison between UV (365 nm, 1560 mW/cm^2^, 5 seconds) and visible light (405 nm, 1650 mW/cm^2^, 5 seconds), irradiation of GelMa encapsulated odontoblast‐like cells with Irgacure 2959 and LAP, respectively, under the similar intensity, cell viability was significantly higher in cells exposed to the higher wavelength.^100^ Interestingly, a marked reduction was observed following 20 seconds of light exposure in the visible light/LAP samples which was not reflected in a UV/Irgacure group which exhibited superior viability for this crosslinking time. Nevertheless, the increased efficiency of LAP suggests that shorter light exposure times can yield sufficiently crosslinked hydrogels hence, the reduced viability observed at longer exposure times does not necessarily have a practical implication. Indeed, a short 10 second exposure time of LAP to UV light (365 nm, 700 mW/cm^2^) produces hardened GelMa‐HAMa scaffolds suitable for cartilage tissue regeneration whereas identical concentrations of other PIs such as Irgacure 2959 and VA‐086 did not achieve the same stiffness even after 2 minutes.^5^, ^62^

The use of visible light (>380 nm) with appropriate PIs can increase the safety of the photo‐crosslinking process by removing exposure to harmful mutagenic light wavelengths, thus maximizing cell viability and reducing the risk of malignant transformation. It has been shown that light at visible wavelength penetrate tissues at higher depths with lower energy compared with UV light, thus making it an efficient source for in situ photo‐crosslinking.^101^ Challenges to visible light crosslinking include the identification of a PI that is sensitive to visible wavelengths as well as identification of the optimal exposure time and power of the light source. Nevertheless, despite showing that the alternative PIs and light wavelength can reduce cell cytotoxicity, the introduction of mutations or DNA damage could not be excluded.

Eosin Y which has a peak absorbance of 530 nm and is being investigated as an injectable material has successfully crosslinked GelMa hydrogels under visible light.^102^, ^103^ Although its use in cell‐laden hydrogels is limited by intrinsic toxicity of the molecule itself, the use of coaxial printing methods which separate cells from the PI may warrant further investigation into the use of Eosin Y and other PIs in tissue engineering. In a recent study, Lim et al investigated the cytocompatibility of human chondrocytes laden GelMa hydrogels fabricated using a set of PIs which absorb in 400‐450 nm of the visible light range: ruthenium (Ru) and sodium persulfate (SPS).^88^, ^104^ The bioscaffolds have comparable physico‐mechanical properties as GelMA photo‐polymerized using Irgacure 2959 and LAP. Moreover, Ru/SPS system has a less adverse effect on the viability and metabolic activity of encapsulated cells for up to 35 days. The major advantages using the Ru/SPS system are significantly higher glycosaminoglycan content and redifferentiation capacity as compared with cells encapsulated using Irgacure 2959 and LAP, and significantly greater light penetration depth compared with the Irgacure 2959 system, allowing thick (10 mm) GelMA hydrogels to be fabricated with homogenous cross‐linking density throughout the construct.

Besides just photoinitiator concentration, light irradiation dose also significantly impacts cytotoxicity and the overall oxidative damage. In their paper, Lim et al showed that even at low PI (Irgacure 2959) concentrations (0.05 wt%), increasing UV irradiation dosage (30 to 50 mW/cm^2^) causes a decrease in cell viability for both human articular chondrocytes and MSCs.^88^

### 6.2 Coaxial extrusion

Given the detrimental effects of free radicals engendered by the photo‐crosslinking process, reducing cell exposure to the PI and its activation products is a possible solution to increase cell viability and protect the DNA molecule from genotoxic effects of free radicals. Coaxial extrusion allows the deposition of cells and the cross‐linking solution through separate internal and external needles, respectively.

This compartmentalization of cells within an inner, non‐crosslinked hydrogel “Core” surrounded by a photo‐crosslinked “Shell” is aimed at protecting vulnerable cells from the toxic effects intrinsic to the PI molecule and more importantly, from free radicals. The shielding effect of cells by the shell compartment increases the scope of materials that can be used for fabricating tissue constructs with a high cell viability. In cartilage tissue engineering, the coaxial method was found to be superior to the monoaxial configuration where cells are embedded throughout the hydrogel and exposed to the toxic photoinitiation process triggered by a fast high irradiance exposure to UV light. In Duchi et al, the cytotoxicity of bioprinted hADSCs upon 10 second irradiation with 365 nm at 700 mW/cm^2^ was found to be negligible, when cells were segregated from the PI, and the viability of UV only irradiated cells was comparable to the nonirradiated control group throughout 7 days.^62^ Moreover, high levels of cell survival immediately after printing and crosslinking with an increase in cell number 10 days following extrusion. In contrast monoaxial printed cells experienced a significant downturn in survival following photoinitiation and a further decrease over time.

## 7 CONCLUSIONS

The hydrogel material and the other additives required to harden the hydrogel itself constitutes the barriers to successful clinical translation of articular cartilage repair techniques using bioscaffolds. The photo‐crosslinking process is an efficient way to achieve a precise spatial and temporal hardening of the hydrogels, but, if not optimized, becomes a major source of cytotoxicity and genotoxicity within the process. These effects are mediated mostly by free radical photoproducts of PI activation, and to a lesser extent, UV light and intrinsic PI toxicity. Cytotoxic effects are regularly evaluated and reported in the literature, but the absence of potentially harmful, tumorigenic DNA lesions has not been proven. With the view of clinical application, safety of the implanted cells inside the bioscaffold is of utmost importance. Therefore, the routine use of standardized techniques to analyze cytotoxicity and genotoxicity, which can be utilized to test products and devices, should be mandatory. Meanwhile, continued investigation into methods of reducing cytotoxicity such as optimization of the photo‐crosslinking conditions or compartment segregation is necessary. Variables within the crosslinking process include PI type and concentration, light wavelength, intensity, and exposure time. Changes to these conditions alter the degree of crosslinking which are directly correlated with the mechanical stiffness of the hydrogel, but also affect cell viability and chondrogenic potential. Hence, both mechanical and biological properties must be considered in the identification of the optimal crosslinking condition. The final goal in the 3D bioscaffold‐based repair of articular cartilage is to deliver a product with enough stiffness to exist within the compressive articular space and support cell survival and chondrogenesis to regenerate healthy cartilage with minimal risk of tumorigenesis.

## CONFLICT OF INTEREST

The authors indicated no potential conflicts of interest.

## AUTHOR CONTRIBUTIONS

C.L., C.O., S.D.: conceived the literature review article, prepared the figures and the tables, and wrote the manuscript; C.D.O., C.D.B., P.F.M.C.: discussed and analytically revised the manuscript.

## Data Availability

Data sharing is not applicable to this article as no new data were created or analyzed in this study.
